# On the significance of estimating cardiorespiratory coupling strength in sports medicine

**DOI:** 10.3389/fnetp.2022.1114733

**Published:** 2023-01-04

**Authors:** Raphael Martins de Abreu, Beatrice Cairo, Alberto Porta

**Affiliations:** ^1^ Department of Physiotherapy, LUNEX University, International University of Health, Exercise & Sports S.A., Differdange, Luxembourg; ^2^ LUNEX ASBL Luxembourg Health & Sport Sciences Research Institute, Differdange, Luxembourg; ^3^ Department of Biomedical Sciences for Health, University of Milan, Milan, Italy; ^4^ Department of Cardiothoracic, Vascular Anesthesia and Intensive Care, IRCCS Policlinico San Donato, San Donato Milanese, Italy

**Keywords:** sports medicine, heart rate variability, cardioventilatory coupling, autonomic nervous system, cardiac control

## Abstract

The estimation of cardiorespiratory coupling (CRC) is attracting interest in sports physiology as an important tool to characterize cardiac neural regulation genuinely driven by respiration. When applied in sports medicine, cardiorespiratory coupling measurements can provide information on the effects of training, pre-competition stress, as well as cardiovascular adjustments during stressful stimuli. Furthermore, since the cardiorespiratory coupling is strongly affected by physical activity, the study of the cardiorespiratory coupling can guide the application of specific training methods to optimize the coupling between autonomic activity and heart with possible effects on performance. However, a consensus about the physiological mechanisms, as well as methodological gold standard methods to quantify the cardiorespiratory coupling, has not been reached yet, thus limiting its application in experimental settings. This review supports the relevance of assessing cardiorespiratory coupling in the sports medicine, examines the possible physiological mechanisms involved, and lists a series of methodological approaches. cardiorespiratory coupling strength seems to be increased in athletes when compared to sedentary subjects, in addition to being associated with positive physiological outcomes, such as a possible better interaction of neural subsystems to cope with stressful stimuli. Moreover, cardiorespiratory coupling seems to be influenced by specific training modalities, such as inspiratory muscle training. However, the impact of cardiorespiratory coupling on sports performance still needs to be better explored through *ad hoc* physical exercise tests and protocols. In addition, this review stresses that several bivariate and multivariate methods have been proposed to assess cardiorespiratory coupling, thus opening new possibilities in estimating cardiorespiratory interactions in athletes.

## Introduction

**FIGURE 1:**
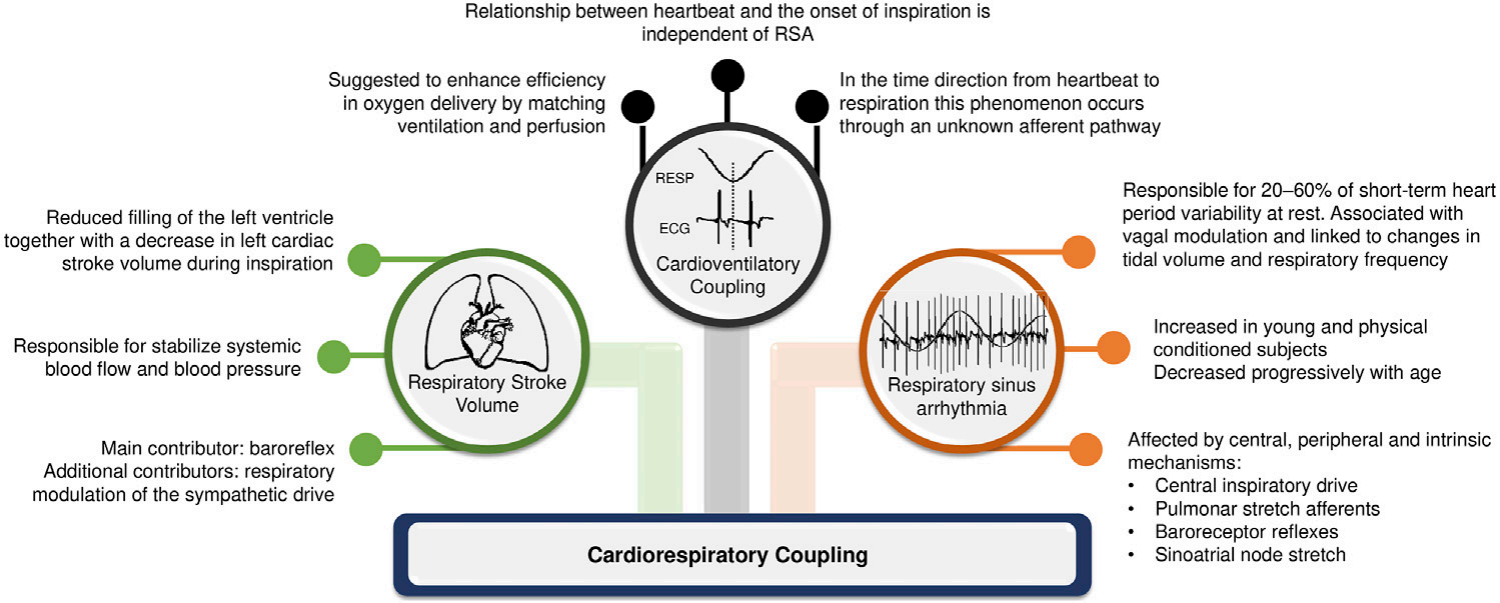
Main mechanisms involved in cardiorespiratory interaction.

The complex interaction between heart and lungs has long been a subject of study since the first recording of respiratory sinus arrhythmia (RSA) by Carl Ludwig in 1847 (Ludwig, 1847). This coupling attracts the interest of experts in different areas of science above and beyond physiology and medicine. In the medical field, the understanding of cardiorespiratory regulation can open new possibilities of clinical interventions. Concomitantly, in the biomedical signal processing field, numerous quantification methods for cardiorespiratory coupling (CRC) assessment have been proposed to account for the complex linear and non-linear interactions between respiratory system and heart, as well as their closed loop relationship with feed-back and feed-forward mechanisms. CRC is related to the neural control of heart period (HP) driven by respiration, with shared inputs, common rhythms, and complementary functions between them (Dick et al., 2014). Although the physiological mechanisms and applications of CRC measurements are still under investigation, some hypotheses have been raised. According to Elstad et al. (2018), there are three types of cardiorespiratory interactions that can determine CRC: respiratory sinus arrhythmia; cardioventilatory coupling; and respiratory stroke volume synchronization (Figure 1).

It is known that HP fluctuates spontaneously according to respiration and its phases (i.e., inspiration and expiration): this physiological phenomenon corresponds to RSA and may partly explain the generation of the CRC pattern (Eckberg, 2009; Dick et al., 2014). RSA describes the modification of the cardiac cycle in relation to respiration, with a shortening of HP during inspiration and a lengthening during expiration, under physiological conditions (Eckberg, 2003). This phenomenon may be determined by neural mechanisms, such as the central influence of respiratory centers modulating vagal motoneuron activity, and changes in intrathoracic pressure and stroke volume during respiratory phases soliciting in the baroreflex (Eckberg, 2003; Eckberg, 2009). During inspiration there is a decrease in intrathoracic pleural pressure which leads to an increase in the pulmonary microcirculation compliance; consequently, venous return is facilitated, which draws blood to the right side of the heart. However, the interventricular septum is mechanically compressed towards the left side, decreasing the left atrial filling. Consequently, left ventricular output drops and so does blood pressure, causing a baroreflex-mediated decrease in HP with the goal of compensating the blood pressure drop (Connolly et al., 1954). In addition, inhibition of vagal motor neurons in the medulla oblongata is expected simultaneously with respiration. During expiration the reverse occurs, with the left stroke volume increasing, so does blood pressure. Consequently, baroreflex and respiratory centers activities modulate the vagal responsiveness, increasing the HP (Eckberg, 2009). To make more complex the interactions respiratory centers can gate vagal activity regardless of the modifications of arterial pressure, namely regardless the intervention of the baroreflex (Eckberg, 2009; Dick et al., 2014) as observed during prolonged periods of apnea.

Several methods to quantify the phenomenon of CRC have been proposed. Traditionally, univariate linear measurements based on frequency domain indexes of HP variability are applied to study cardiac autonomic regulation and especially related to the activity of its parasympathetic branch. The high frequency (HF, from 0.15 to 0.4 Hz) power expressed in absolute units, usually ms^2^, is commonly utilized to estimate the influence of respiration on cardiac neural regulation (i.e., RSA). However, considering the complex interactions of the subsystems abovementioned and the potential role of baroreflex mediation, a network physiology approach might be better suited to CRC assessment, especially whether the analysis aims at ruling out the influence of confounding factors. The network physiology is particularly appropriate to study complex interactions at multiple levels of integration among regulatory mechanisms and organs (Ivanov, 2021). In CRC analysis, a network physiology approach can favor the description of the neural network at the basis of the coupling between HP and respiration, as well as how it can be modulated, for example, by physical exercise.

Therefore, the computation of the CRC must consider at least a bivariate analysis framework (i.e., HP variability and respiration), although the inclusion of systolic arterial pressure (SAP) to the model as a confounding factor might also be necessary to account for influence the baroreflex (de Abreu et al., 2020; Porta et al., 2012). Linear models may also be insufficient to quantify the complexity of physiological subsystems that interact in a non-linear way (Hayano and Yuda, 2019) and directionality of interactions between biological signals (e.g., from respiration to HP series) should be considered for a better understanding of causal cardiorespiratory relations (Porta et al., 2013b; Schulz et al., 2013; de Abreu et al., 2020). Furthermore, it has long been established (Penzel et al., 2016; Elstad et al., 2018) that CRC is a complex phenomenon encompassing different forms of interaction between the two systems, which are not always observed concomitantly in the same experimental condition and population (Schäfer et al., 1998).

It is known that cardiac and respiratory systems have multiple forms of coupling which can simultaneously coexist at different time scales, with intermittent “on” and “off” periods. Therefore, in addition to the traditional RSA measures, quantifying cardio-respiratory phase synchronization (CRPS) could be important to determine the degree of CRC, while time delay stability (TDS) analysis can allow the monitoring of the delay of the closed loop relationship between the HP and respiration (Bartsch et al., 2012; Bartsch et al., 2014). Measures of CRPS and TDS might open multiple windows on CRC by providing description of peculiar aspects of the phenomenon that cannot be completely described by traditional markers such as respiratory rate and levels of autonomic modulation (Bartsch et al., 2014).

The computation of the CRC indexes seems to be useful in the sports medicine and is strongly affected by physical exercises. There is a hypothesis that the increase in CRC is associated with efficiency of gas exchange by matching pulmonary perfusion to ventilation during inspiration, which may be associated with higher VO_2peak_ values, since VO_2peak_ is determined in part by a great efficiency in gas exchange (Dick et al., 2014; Hayano and Yuda, 2019; Abreu et al., 2022a). For example, it is known that RSA increases after a high-intensity training period in runners (de Meersman, 1992) and cyclists (Al-Ani et al., 1996), alongside a significant increase in VO_2peak_. In (Perry et al., 2020, 2019) an increase of CRPS was found both in athletes engaging in high-intensity training that encourage controlled, rhythmic breathing and in individuals performing other less rhythmic forms of exercise.

In line with these findings, a recent study showed that cyclists have greater CRC strength compared to sedentary individuals (Abreu et al., 2022b). Moreover, this difference was identified only when non-linear approaches were applied to compute the CRC, such as joint symbolic analysis (Abreu et al., 2022a). In addition, an increase in CRC was detected during exposure to intense aerobic exercise under hypoxic situations (Uryumtsev et al., 2020). Conversely, a decrease in CRC strength was associated with situations evoking a high sympathetic tone (Iatsenko et al., 2013). Indeed, CRC decreased progressively during graded head-up tilt (Porta et al., 2012).

This review explores the relevance of assessing CRC in sports medicine by listing a series of applications in athletes and providing some suggestions about the methods that can be utilized for its quantification.

## Bivariate and multivariate CRC assessment applied to athletes

Methods for the estimation of CRC in athletes are summarized in Table 1. A first interest in CRC evaluation in athletes was shown by Schafer et al. (1998), Schafer et al. (1999) who evaluated CRPS in high performance adolescent swimmers *via* the original methodology of the synchrogram (Schäfer et al., 1998). This technique computes the phase of the occurrence of each heartbeat with respect to inspiratory onsets. Phases are plotted as a function of the number of cardiac beats. Epochs of synchronization appear as *n* horizontal lines in the graph. Analysis can be made more complex by grouping *m* consecutive respiratory cycles and assessing the phase of each heartbeat with respect to onset of first respiratory cycle, thus distinguishing more complex *n*:*m* phase locking from simpler *n*:1 ratios, where *n* is the number of heartbeats detected at repetitive phases within *m* respiratory cycles. Advantages of synchrogram analysis are that this approach accounts for non-linear regimes of CRPS and can distinguish within the same recording multiple periods of CRPS at different *n*:*m* ratios, without any *a priori* assumptions. Since the seminal studies that applied synchrogram in swimmers (Schäfer et al., 1998; Schäfer et al., 1999), the methodology was applied in Ironman athletes (Angelova et al., 2021), amateur cyclists (Cairo et al., 2021), and in protocols of paced breathing at very high breathing rate (Perry et al., 2019; Perry et al., 2020). Metrics utilized for quantification of synchrogram in athletes range from the total length of synchronization periods at *n*:*m* ratio (Schäfer et al., 1998; Schäfer et al., 1999; Angelova et al., 2021) and the averaged synchronization index with assigned phase locking ratio and fixed threshold for synchronization detection (Perry et al., 2019; Perry et al., 2020) to the percentage of synchronization windows *via* automatic threshold optimization (Cairo et al., 2021).

**TABLE 1:** Review of methodologies used in CRC assessment in athletes.

Considered variables	Method	Applications	Limitations
Bivariate	Synchrogram Schäfer et al. (1998)	Swimmers Schäfer et al. (1999), Schäfer et al. (1998), Ironman athletes under physical and cognitive stress Angelova et al. (2021), amateur cyclists undergoing inspiratory muscle training and postural challenge Cairo et al. (2021), and protocols of paced breathing at very high breathing rate Perry et al. (2019), Perry et al. (2020)	The method does not account for directionality of interaction
	Distribution of time intervals between respiratory events and heartbeat Galletly and Larsen (1997)	Amateur cyclists undergoing inspiratory muscle training and postural challenge Cairo et al. (2020)	The method considers only the heartbeat immediately preceding or following respiratory events, regardless of the rest of the heart rate variability series
	Joint symbolic analysis Porta et al. (2015)	Amateur cyclists and non-athletes undergoing an orthostatic stressor Abreu et al. (2022a)	The method does not account for directionality of interaction
	Squared coherence Saul et al. (1991)	Amateur cyclists undergoing an orthostatic stressor de Abreu et al. (2020), and male runners in hypoxic conditions Uryumtsev et al. (2020)	The method does not account for temporal directionality and non-linear dynamics
	Granger causality Granger (1963), Porta and Faes (2016)	Olympic athletes and non-athletes undergoing postural challenge Młyńczak and Krysztofiak (2019)	The method does not account for non-linear dynamics
Multivariate	Conditional granger causality and transfer entropy Granger (1980), Barnett et al. (2009)	Amateur cyclists undergoing an orthostatic stressor de Abreu et al. (2020)	The method does not account for non-linear dynamics
	Principal component analysis Daffertshofer et al. (2004)	Aerobic and resistance training protocols Balagué et al. (2016), maximal exercise test Garcia-Retortillo et al. (2017), high-intensity interval training and moderate-intensity training during maximal exercise test Garcia-Retortillo et al. (2019), and acute hypercapnia in mountaineers Gultyaeva et al. (2021)	The method does not account for temporal directionality and non-linear dynamics

However, synchrogram analysis does not account for directionality of the interactions: conversely, a researcher might be interested in distinguishing non-linear interactions that are due to the influence of respiratory system on the heart from those in the reverse causal direction given that both the directions are found to be relevant (Porta et al., 2013a; Penzel et al., 2016). As such, it is necessary to employ different methodologies of CRC assessment that can account for temporal precedence of the activity of one oscillator over the other. A method taking into consideration the directionality of the interaction was proposed by Galletly and Larsen (Galletly and Larsen, 1997) who computes the distribution of time intervals between a respiratory event (i.e., inspiratory, or expiratory, onset) and the heartbeat immediately preceding, or following, the event itself. The normalized Shannon entropy of the distribution is taken as a measurement of directional coupling (i.e., from respiratory system to the heart and *vice versa*). Cairo et al. (2020) applied to amateur athletes the method proposed by Galletly and Larsen (Galletly and Larsen, 1997) and found a decrease of cardiorespiratory coupling after sympathetic activation induced by active standing in both causal directions. Remarkably, the entity of the observed decrement depended on the intensity of training (Cairo et al., 2020).

Another methodology for CRC assessment utilized in athletes is the joint symbolic analysis applied to the beat-to-beat variability series of HP and respiration (Abreu et al., 2022b). Specifically, the two beat-to-beat HP and respiratory series undergo a separate symbolization procedure and short patterns of each series are classified according to their temporal dynamics (Porta et al., 2001). The percentage of occurrence of joint schemes of coordinated HP and respiratory patterns can describe the strength of CRC at different time scales, without considering their directionality (Porta et al., 2015). Furthermore, the method is capable of accounting for non-linear dynamics (Guzzetti et al., 2005; Porta et al., 2015) present in the cardiorespiratory system.

In the spectral domain, the squared coherence function between the beat-to-beat variability series of HP and respiration, can be used to evaluate CRC. Squared coherence function is defined as the ratio between the squared cross-spectral density between HP and respiration divided by the product of the power spectral densities of the two series (Saul et al., 1991). Squared coherence is usually averaged in specific frequency bands. Remarkably, both low frequency (LF, 0.04–0.15 Hz) and HF have been utilized in athletes according to the application: the LF band was utilized in hypoxic conditions in male runners (Uryumtsev et al., 2020), and the HF band in an inspiratory muscle training (IMT) protocol in amateur cyclists (de Abreu et al., 2020). The most important drawback of this method is its inability of accounting for temporal directionality and non-linear dynamics.

Causal methods utilizing the Granger causality framework (Granger, 1963; Granger, 1980) have been applied to CRC assessment to distinguish between Olympic athletes and non-athletes (Młyńczak and Krysztofiak, 2019). Briefly, according to the Granger causality framework a variable is taken as the presumed cause of an effect variable if a predictive model of the future of the effect based on the past values of both variables is more efficient than a model based solely on the past values of the effect variable. In (Młyńczak and Krysztofiak, 2019), pairwise-conditional, spectral, and extended Granger causality (Porta and Faes, 2016) were used to investigate the directional links between the beat-to-beat HP and respiratory series, or the breathing phases series. Granger causality approach have been turned into the information domain as well, *via* the concept of transfer entropy, namely the reduction of the information carried by the target (here HP), above and beyond the contribution of past HP values, when the driver (here respiration) is included in the model, (Schreiber, 2000; Barnett et al., 2009). Measures of Granger causality, including transfer entropy, can account for confounding factors by considering additional drivers in the model. Transfer entropy was applied in the CRC assessment of amateur cyclists undergoing an orthostatic stressor (de Abreu et al., 2020). Remarkably, in Abreu et al. (de Abreu et al., 2020) the SAP beat-to-beat series was considered as a confounding factor to exclude the cardiac baroreflex influence on the cardiorespiratory link.

An additional multivariate methodology that has been employed for CRC assessment in several studies (Balagué et al., 2016; Garcia-Retortillo et al., 2017; Garcia-Retortillo et al., 2019; Gultyaeva et al., 2021) is the principal component analysis (PCA) between cardiovascular time series (i.e., HP and arterial pressure) and respiratory parameters (i.e., expired fraction of O_2_, expired fraction of CO_2_, end-tidal partial pressure of oxygen, end-tidal partial pressure of carbon dioxide, ventilation). PCA is a statistical technique of dimensionality reduction (Daffertshofer et al., 2004), frequently used in coupled systems with multiple degrees of freedom and complex dynamics, which minimizes the number of co-varying components, while accounting for the majority of the information contained in the original multivariate dataset. The methodology has been tested in a variety of applications: aerobic and resistance training protocols (Balagué et al., 2016), maximal exercise test (Garcia-Retortillo et al., 2017), high-intensity interval training and moderate-intensity training during maximal exercise test (Garcia-Retortillo et al., 2019), acute hypercapnia in mountaineers (Gultyaeva et al., 2021).

## CRC measures in sports medicine

The strength of CRC varies in athletes when compared to age-matched sedentary individuals, both at rest and during stressful stimulus (Abreu et al., 2022a). A study conducted with recreational cyclists, who trained for at least 6 uninterrupted months, at least 150 min a week, showed greater CRC strength during spontaneous breathing at rest and after orthostatic stress (i.e., active postural maneuver), when compared to individuals who did not perform any exercise (Abreu et al., 2022b). In addition, the study identified an increase in vagal modulation directed to the sinus node assessed through spectral analysis of HP variability, although this finding may be controversial in the literature. Possibly confounding factors may lead to misinterpretations of the estimation of cardiac vagal modulation when considering univariate measures, such as the HF component of the HP series (Hayano and Yuda, 2019). These misinterpretations are likely to be linked to the arbitrary limits of the HP band in connection with the complexity of the regulation of the tonic value of vagal activity with respect to its changes responsible for RSA (Hayano and Yuda, 2019). Therefore, computing the CRC by considering at least a bivariate approach that includes the recording of respiratory signal might favor a more genuine measure of CRC. In addition to greater CRC strength, amateur cyclists also had lower respiratory rate values when compared to sedentary individuals at rest and during orthostatic stress, thus suggesting that respiration exerts an important influence on the regulation of spontaneous cardiovascular dynamics, as well as during stressful stimuli (Abreu et al., 2022a).

CRC measurements can also be useful to identify autonomic responses to different modalities of physical training. A study evaluating the CRC of athletes undergoing an IMT protocol identified that 11 weeks of moderate-intensity IMT may favor cardiac autonomic control due to chronic modifications on the CRC (de Abreu et al., 2020). Changes in CRC also favored better adjustments to active postural maneuver, regardless of cardiac baroreflex regulation. Although the physiological mechanisms involved are not fully elucidated, hypotheses suggest that IMT can promote changes at the central respiratory network level through the repetitive activation of pulmonary and atrial afferent stretch receptors (de Abreu et al., 2020; Eckberg, 2009). Chronically, IMT can affect cardiac autonomic discharge driven by respiratory centers as well *via* periodical solicitations of the baroreflex control, thus favoring a more efficient response to standing (DeLucia et al., 2018).

In middle-distance runners, CRC markers proved to be important for understanding the mechanisms of oxygen supply during intense physical activity, as well as they were utilized as additional signs for the prognosis of qualification level in runners (Uryumtsev et al., 2020). CRC changes were evaluated in high- and moderate-level runners in response to exposure to a hypoxemic mixture of 10% O_2_ content through a face mask. It is known that acute hypoxemic exposures cause an increase in sympathetic activity and sympatho-respiratory coupling, as a result of a synchronized activation of respiratory and sympathetic medullar neurons (Zoccal, 2015; Lindsey et al., 2018). In high-level runners, the response to hypoxemic exposure was accompanied by a decrease in oxygen consumption and an increase in CRC strength. The authors suggest that high-level athletes have better integration of subsystems to cope with the hypoxemic response and that the CRC probably plays an important role of tuning and synchronization of rhythms to save energy, which explains the reduction in oxygen consumption (Moser et al., 2006). Therefore, strengthening CRC integration is an adaptative adjustment of cardiorespiratory system to provide an optimal response to hypoxia, especially in athletes during intense aerobic training.

In Ironman athletes, CRC was investigated pre and post Ironman event in order to determine the effects of pre-competition stress on the cardiac and respiratory systems (Angelova et al., 2021). The CRC acquisition was performed during a cognitive task (Stroop test) to avoid the athletes from cognitively controlling their respiratory frequency. The authors found that CRC values increase post-competition when compared to pre-competition, suggesting that the amount of stress the athletes are recovering during post-competition session is greater than effects of cognitive stress test as a likely sign of vagal posr-exercise rebound. Moreover, CRC coordination was found to be important during recovery from an intense effort and remains active during a cognitive task (Angelova et al., 2021).

## Conclusion

The interest in CRC investigation and its applicability has grown in recent years. The present review showed that CRC can be a valuable and non-invasive tool for studying the neural interaction between respiration and cardiac activity in the field of sports medicine, although several gaps still must be filled. Increases in CRC seem to be a physiological mark of aerobic physical training, being associated with the level of qualification of the athletes, as well as post-competition recovery capacity and adaptations to hypoxemic environments. Furthermore, exercise modalities such as IMT may be an option to optimize CRC responses at rest and during physiological stress in cyclists. Although CRC strengthening is associated with positive physiological outcomes, future studies should investigate the impact of CRC on sports performance assessed through physical exertion tests, as well as the effects of different training modalities on these indexes. In addition, sports professionals should be aware of various possibilities to estimate the CRC provided by modern biomedical signal processing.
